# Quantitative and qualitative analysis of the knowledge, attitudes and social representations of cholera in the extreme northern region of Cameroon: the case of Maroua I, Maroua Ii And Mokolo

**DOI:** 10.11604/pamj.2014.17.253.2799

**Published:** 2014-04-08

**Authors:** Penn Amaah

**Affiliations:** 1 International Relations Institute of Cameroon (IRIC), Yaoundé, Cameroon; 2 Njikwa-Oshie Sub-divisional Hospital, North West region, Cameroon; 3 Faculty of Medicine, Biomedical sciences, University of Yaoundé I, Yaoundé, Cameroon

**Keywords:** Cholera, social representations, intercultural dialogue, intercultural mediation, participative research, constructivism

## Abstract

**Introduction:**

An effective fight against cholera requires an in-depth consideration of the knowledge, attitudes and social representations of cholera within a population. Cholera outbreaks persist in the Extreme North of Cameroon because of the inadequate integration of representations of cholera, water and hygiene in the fight against this disease. Through a constructivist intercultural approach not conflicting with the western ethnocentric model, socio-cultural/religious and historical ideologies can be reconciled to provide optimal and sustainable healthcare solutions to the repeated and long lasting cholera epidemics using participative research, intercultural mediation and dialogue in Cameroon.

**Methods:**

Through a cross-sectional, ethnographic and participative study, data was generated using semi-directed in-depth interviews of key informants, collection of videos, pictures and the completion of 2 pre-tested questionnaire types in 3 communities (Maroua I, Maroua II and Mokolo). Quantitative data was entered using Ms Excel and Epi Info 7, and analysed using Epi Info 7. Qualitative data was analysed inductively using the concept of social representations.

**Results:**

Results show evidence of the inadequate integration of cultural and socio-cultural factors favouring cholera spread and a respondent population majority unable to identify this (92.82%). Equally identifying environmental and cultural factors, the results bring out the impact of the on-going cholera combating strategy.

**Conclusion:**

Representations of cholera, cultural and socio-cultural values are not adequately considered in the fight against cholera. We recommend policy-makers and health actors to improve on the integration of these through advocacy, in designing, communicating and implementing effective prevention strategies via participative research, intercultural mediation and dialogue.

## Introduction

Social representations in the contemporary world remain part of a global means to understand and provide solutions to the world's multitude of problems regarding health, the environment and human development using a method of participative research. Underpinning both thoughts and actions, social representations respond to a reality which is collectively and socially constructed, logically bringing meaning to assumptions/explanations people give to social phenomena [1]. They provide a useful framework by showing how socio-cultural and historical forces impact on the health of a group of people. According Denise Jodelet and her work on psychoanalysis, the importance of social representations lies in the interface between social phenomena and psychology [2].

Unfortunately in post-colonial Africa today, a majority of experts still execute health and development projects based on a western ethnocentric model. The fruits of such an approach remain short-lived and unsatisfactory as each country, society and locality has a specific language which is part of her social representation and cultural identity. This clearly has a direct or indirect effect on the approach which is used. Public health officials and other professionals working in the health sector require a mastery of social representations that a particular population or individual may possess with respect to a particular disease. The African continent with a plurality of cultures has a plurality of logics and visions of its respective societies in the perception of health, disease and development issues [3]. Thus, there is the need to employ the concept of social representations as a methodology to integrate the cultural realities, know-how and savoir-faire of populations in the planning of prevention, treatment and monitoring schemes using a socio-constructivist approach.

In the light of our reflection on the rampant cholera epidemics in Cameroon, it is highly recommended to know and understand public opinion about the causes of this infectious disease in order to formulate, communicate and put in place effective and participative cholera eradication programs that respect culture in affected localities. Cholera transmission which is closely linked to inadequate sanitation and environmental management can also occur as consequence of a disaster disrupting water and sanitation systems, displacing populations to inadequate and overcrowded camps [4]. According to the WHO Global Task Force of Cholera Control, Cameroon has been hit by at least 10 cholera epidemics since 1971 with a progressive increase in thousands of those affected [5]. The case fatality rates have ranged between 3.8% and 15%. The Weekly Epidemiological Record (WHO, 2011) reports 22433 cases with 783 deaths and a case fatality rate of 3.49% [4]. This same report shows a doubling of the reported cases in the same region between the years 2010 and 2011. The same statistics reveal that this epidemic lasts long and is on the rise with deaths of thousands of those affected. The recent epidemic in the North of Cameroon from 2009 to the end of 2011 is the most severe of the cholera outbreaks in the history of the region. Globally, WHO reports a hike in the number of cases of cholera infection. From 2004 to 2008, cases have increased by 24% compared with the period from 2000 to 2004. The true burden of the disease is estimated at 3–5 million cases and 100 000–120 000 deaths annually.

The general objective was to ameliorate on the fight against cholera in evaluating the knowledge, attitudes and social representations of the population with respect to cholera, hygiene and water in the Mokolo, Maroua I and II localities. Specifically, it sought to identify what cultural and socio-cultural factors influenced the fight against cholera and other water borne diseases, to explain how particular positive aspects of culture could be promoted in the fight against infectious disease spread, to bring out the impact of the on-going cholera combating approach on the Extreme Northern culture, to identify environmental and social factors encouraging the persistence of cholera and finally, to evaluate the knowledge of the population on basic hygiene.

## Methods

**Study design and Setting:** The study running for 9 months was cross-sectional, ethnographic and participative in nature carried out in the localities of Mokolo, Maroua I and II in the Extreme Northern region of Cameroon.

**Sampling and data collection method:** Communities were divided into 5 clusters each and homes were randomly selected from each cluster. Two pre-tested questionnaire types and cameras were used to collect data while taped semi-directed interviews were used to extract information from key informants and community personnel on the lifestyles, water treatment and waste disposition schemes of the population. The questionnaires were administered to patients/clients visiting the hospitals, to households, health personnel and to key informants of communities. An individual from each randomly selected household answered the questionnaire. Responses from clients visiting public hospitals and clinics in this region were included. A total of 237 persons responded to the first questionnaire, 16 persons to the second questionnaire and 5 key informants were semi-directly interviewed. Open ended questions were used to permit the respondents have the opportunity to actively propose their answers rather than choose from a passive range of displayed choices.

**Data entry and Analysis:** Quantitative data was entered using Ms Excel 2010 and Epi Info statistical package 7, and analysed using Epi Info. Qualitative data was analysed inductively using the concept of social representations and was based on reflections following the observation of images, attitudes, ways of living and the environment in the communities in question.

**Administrative authorization:** The study was reviewed and approved by the International Relations Institute of Cameroon. Administrative clearance was obtained from the Extreme North regional delegation of public health.

## Results

### Respondent Demographic data

**Figure 1:**
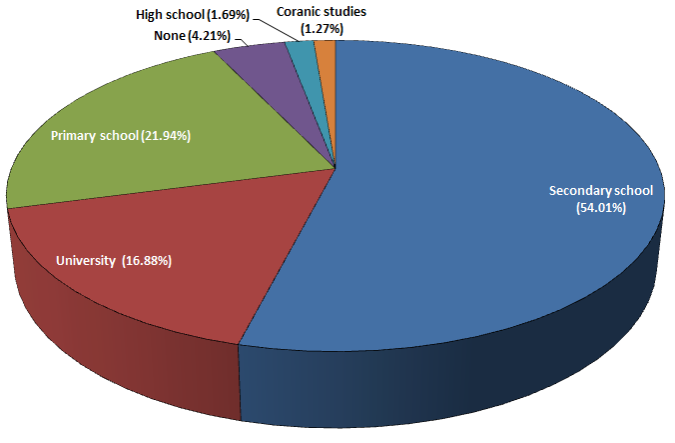
Distribution with respect to the highest educational level

**Population Distribution:** Table 1 shows the distribution of the 237 respondents of the first questionnaire from the 3 communities. The ages of the respondents of the first questionnaire type ranged between 12 and 55 years, with a mean age of 26.22 ± 7.47 years. A majority of the respondent population was of the Muslim faith (85.23%) as opposed to Christians (14.77%). Figure 1 divides the population according to their highest educational level attained.

**Table 1:** Population and sex distribution amongst the different recruitment communities

	Maroua I	Maroua II	Mokolo	Total
Male	68 (28.69%)	87 (36.70%)	58 (24.47%)	213 (89.87%)
Female	11 (4.64%)	8 (3.38%)	5 (2.11%)	24 (10.13%)
**Total**	**79 (33.33%)**	**95 (40.08%)**	**63 (26.58%)**	**237**

**Population distribution with respect to occupation:** a majority of the respondents were unemployed (29.11%). Animal breeding (18.98%) and involvement in local business ventures (15.18%) were common occupations. Farming, vending, driving, photography and house wives were other occupations cited.

**Knowledge of cholera:** although all the respondent population had heard of cholera, only 2.53% admitted to have suffered from a cholera infection. Accepting to know someone who had suffered from cholera were 56.54% of the respondents.

**Means of contracting cholera:** contaminated food (86.07%) and water (77.21%) were the highest responses cited as means of contracting cholera. Another 1.26% cited other relevant causes (dirty environment, flies, poor hygiene and failure to clean hands) and some 4.64% didn't know how cholera is contracted.

**Knowledge of signs and symptoms of cholera:** some 17.30% of the respondents couldn't cite any sign or symptom of cholera. Of the total knowledgeable respondents (82.70%), 6.12% still cited a wrong and non-specific symptom ‘rise in body temperature’. With regards to identifying signs and symptoms of cholera, respondents predominantly cited diarrhoea (76.02%), vomiting (55.10%) and weight loss (11.73%).

**What do you do to treat yourself if you suspect you have cholera?:** when suffering from cholera, a majority will go to the hospital (97.46%). In addition to seeking medical care, 11.39% will pray. Some 4.60% admitted to seeking traditional medicine and 4.60% opting for auto-medication by buying and consuming road side drugs.

**What do you do to prevent cholera in your community?:** answers forwarded as propositions for the prevention of spread of cholera were: 83.54% opting for respect of the rules of hygiene, 5.90% citing a reduction in the overcrowding of people, 79.74% opting for sensitization on the proper use and maintenance of latrines. With regards to the causes of cholera, 98.73% of the study population identified dirtiness as a major cause. However, 2.53% of the total population mentioned lack of education as a factor favouring its spread.

### Attitudes with respect to cholera

**Figure 2:**
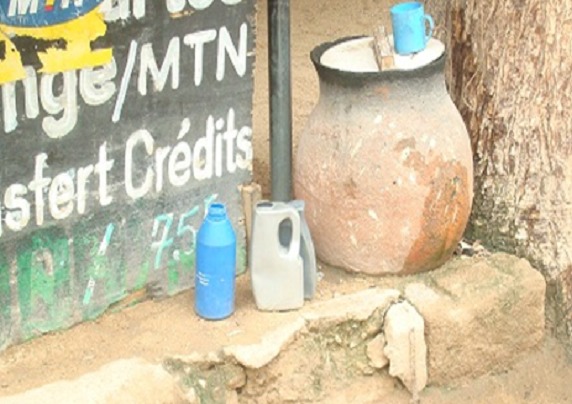
Image of a road side water stand (canari) with a cup under a tree

With 92.82% of the respondent population denying the presence of Extreme-Northern cultural practices that encouraged/influenced the spread of cholera, only 7.17% cited cultural practices/habits encouraging the spread of cholera such as: the habit of eating in groups observed in families, drinking from local road side public drinking water stands (Figure 2), poor hand washing habits in restaurants, poor drinking habits/environment of the population in bilibili cabarets(locally produced drink form millet) and the unhygienic circumstances surrounding bilibili production, the non-use of latrines, the presence of animal faeces and the practice of sitting in the sand. Other factors fuelling dissatisfaction and breach of cultural values were; the stripping naked of infected women in front of male medical personnel and the prohibition of visitors from meeting the sick. Some 8.01% of the population still attribute cholera infection to mystic causes (witchcraft). Although a majority (84.81%) of the population were satisfied with the approach used by the hospital (Ministry of Public Health) in the management of this disease, 15.19% remained unsatisfied attributing their dissatisfaction to the numerous deaths that were registered in hospitals. However, none of the respondents would accept to use non-medical treatment for the management of cholera cases contrary to previous questions and all accepted that the fight against cholera required community effort. With regards to the attitude of respondents towards to those who have suffered from cholera, 29.53% of the population affirmed having a stereotyped view. They attributed to the latter words such as; resentment, stigma, victims of witchcraft, carriers, negligence, mockery and dirt. When asked what to do when a person dies of cholera, only 53.58% of respondents gave meaningful suggestions. Their suggestions included; avoiding the manipulation of corpses, disinfecting the environment and nearby personnel, burning the dresses of the dead, alerting medical personnel, digging a very deep hole at the burial site, wearing of gloves and other protection material when carrying corpses, and the use of antiseptic to wash hands.

### Practices linked to cholera

All respondents affirmed having toilets and using them regularly. Their toilet types varied between traditional latrines (64.97%), water closet (31.64%) and 3.39% had both toilet types. Some of the respondents’ (46.41%) reasons for constructing external toilets were; to do away with bad odours, to keep away flies from the house, because of water scarcity to run a water closet system, to respect a local custom of constructing toilets outside homes and to facilitate latrine access to strangers. Some 9.28% of respondents bathed in streams and rivers finding reason for this in the fact that water was scarce or they were doing so for recreation purposes. However, 87.34% affirmed seeing children and adults bathe in local streams and rivers. Of the respondent population, only 56.96% had access to potable water using the CamWater (Cameroon Water Utilities Corporation) and fountain water as sources. The rest (32.35%) did not sterilize water before consumption. All respondents washed their hands before meals, although only 73.83% would use a detergent.

### Responses of medical and paramedical personnel (second questionnaire)

Some 16 medical personnel answered this second questionnaire type. All respondents in this category denied knowledge of the use of traditional medicine by the population in the treatment of cholera. The principal fears of those who suffered from cholera infection were death (100%) and stigma (30%). Medical and paramedical personnel reported the fear of the population in transporting contaminated sick patients to the hospital for fear of contamination. Another reported difficulty at the level of the hospital was that of quarantining infected patients. Family members and well-wishers often visited to see the sick even when they were advised not to. According to medical and paramedical personnel, dirt, witchcraft and food poisoning were common perceptions among the population of why cholera was contracted. Cited as a positive cultural/religious habit that could be implemented to combat the propagation of cholera was the modification of the hand washing practice before prayers in this Muslim community using a detergent. The respondent population proposed the use of a detergent especially during epidemics. Also mentioned in this category of respondents was; eating in groups, the poor use/lack of latrines, drinking from road side canaris and poor hand washing habits before prayers as cultural/religious habits that could favour the spread of cholera.

## Discussion

Cholera (locally called *Nya-o salute*) remains a persistent health problem faced by the Extreme Northern region of Cameroon given its flat topography, climate and culture. The risk of having more epidemics, increases morbidity and mortality because of identified cultural and socio-cultural factors such as; dipping a common un-rinsed cup and drinking from local road side water vessels (canaris), eating in groups after washing hands usually in a common bowl of water without changing it as observed in families, reported hand-washing of the perineum after defecation among some members of this community, poor hand washing practices observed in restaurants, and poor hygienic circumstances surrounding local drinking spots (cabarets) including the dirty environment, the presence of flies(vectors of disease) and the habits of drinkers.

Despite the presence of the above factors, a majority of the population (92.92%) either denied or couldn't identify cultural practices influencing cholera spread and 8.01% still attributed unnatural causes(mysticism or poisoning) to infection with cholera. Although the Ministry of Public Health and International NGOs play a major role in fighting cholera, some 15.19% of the respondent population were not satisfied with their combat strategy. They decried the stripping naked of infected female patients in front of male medical personnel and the prohibition of visitors from meeting the sick. This just confirmed their limited knowledge and understanding as 4.64% of the respondents didn't know how cholera was contracted, 6.12% citing a wrong and non-specific symptom “rise in body temperature” and only 53.58% giving positive suggestions on what to do when someone dies of cholera. About one-third (29.53%) of the respondents had a biased view of treated cholera patients using words such as stigma, dirt, victims of witchcraft amongst others.

Even though the landscape of the Extreme Northern region is flat, there are no efforts to construct drainage patterns. Existing ones are poorly maintained and blocked with dirt. The arid climate favours the growth of flies which are vectors of water borne diseases. Also present are plant species attracting a lot of flies during their flowering season. Due to water shortages, only 56.96% of respondents have access to potable water sources. Some members of the community use water from nearby streams and rivers in which children and adults are often seen bathing or washing clothes.

While examining important neglected factors to be considered for sustainability in the fight against cholera, the study shows particular characteristics of the study population. However, it fails to explore the best communication channels needed to reach the population with information. Information gathered here can't be generalized to other regions affected by cholera in Cameroon.

Compared to an Extreme Northern provincial quantitative study in 1997 on the knowledge, attitudes and behaviours with respect to cholera [6], this paper has strengths and weaknesses. The 1997 study concentrated just on knowledge, attitudes and behaviours. It failed to explore the representations of cholera as a disease, the perception of cholera and the populations’ satisfaction with regards to the approach that was used. The previous did not bring out the point of view of the health personnel with regards to the combat of cholera and their perception amongst the population. The 1997 study however was exhaustive covering all divisions of the Extreme Northern region of which the current did not. This paper did not explore the best communication means to reach the population, whereas the previous did. It remains possible that the method of communication could have changed given the advances in communication and technology over the past fifteen years.

A majority of the respondents heard of cholera and more emphasis should be laid not only on hygiene but on drinking/eating habits. Preventive measures should centre on group meals and hand washing, manipulation of water at the roadside vessels (canaris) before drinking, perineal hygiene, habits of clients in restaurants and local drinking spots (cabarets). Sensitization should reveal the presence of cultural factors encouraging cholera spread while proposing intercultural reconciliatory means that preserve local cultural values. It should integrate messages on negative representation demystification of cholera infection and make it clear that infection is not unnatural or mystic but related to infection, poor hygiene, bad and risky habits. Messages should be clear and precise to do away with prejudice and stigma attributed to victims of cholera infection in the society. Information should equally carry messages on what to do during an epidemic or when one comes across an infected individual. The Ministry of Public health should demonstrate gender sensitivity in deploying human health resources in this part of Cameroon. Local councils should be encouraged to keep their localities clean and unblock existing drainage patterns to ease the flow of water in order to prevent stagnation of water after rainfall. They should also devise means to multiply potable water sources to the population while devising a means of controlling the quality of water at the roadside water stands.

The significance of this study lies in identifying obstacles and proposing ways to ameliorate the fight against cholera epidemics in the Extreme Northern region of Cameroon. The findings are resourceful to policymakers in the design, formulation, advocacy and communication of effective cholera fighting strategies and policies. The discussion advances sustainable solutions to identified negative representations and proposes a flexible combat approach which permits the implementation of the western combat model while respecting the local culture and customs of the people.

An important but unanswered question to be studied is the best channel to communicate information in this Muslim community. For a positive impact, this needs to be well understood. Further research should also cover all other cholera affected areas in Cameroon since the current findings are specific and do not necessarily apply to them. For sustainability and efficiency reasons, pilot surveys should assess projects based on the sustainable development four-pillar model.

## Conclusion

Health and health partners in Cameroon are not adequately considering representations of cholera, cultural and socio-cultural values in the fight against cholera. This in part explains the persistence and repeated epidemics in the Extreme Northern region. Campaigns should be fortified to include brief convincing messages on roadside drinking water vessels, hygiene in drinking spots/restaurants, eating in groups and perineal hygiene, using appropriate communication strategies effective in this population. Gender sensitivity should be demonstrated in the deployment of human health resources. Mediation should be multi-sectoral, educational and cultural planned together with the help of health structures, local councils and mosque/church/school/community leaders for a better results.

## References

[1] Elame E (2010). Les Représentations sociales comme méthodologie de recherche action participative dans les politiques de développement, Master, Coopération Internationale, Action humanitaire, et Développement durable. Centre Inter-Universitaire pour la Recherche Didactique et la Formation Avancée.

[2] Jodelet D (1991). L'idéologie dans l’étude des Représentations Sociales in Aesbischer V, Deconchy JP, Lipiansky R, Idéologies et représentations sociales.

[3] Mbonji Edjenguèlè (1998). Les Cultures de développement en Afrique. Essai sur l'impossible développement sans révolution culturelle.

[4] World Health Organization (2011). The Weekly Epidemiological Record on Cholera. http://www.who.int/wer/2012/wer8731_32.pdf.

[5] World Health Organization (2012). Global Task Force for the Control of Cholera. http://www.who.int/cholera/countries/CameroonCountryProfile2011.pdf.

[6] CARE (1997). Enquête quantitative de base sur les connaissances, attitudes et comportements des populations de l'Extrème Nord vis-à-vis du cholera. http://www.minsante-cdnss.cm/download/file/fid/269.

